# Dynamic Formation of a Posterior-to-Anterior Peak-Alpha-Frequency Gradient Driven by Two Distinct Processes

**DOI:** 10.1523/ENEURO.0273-24.2024

**Published:** 2024-08-23

**Authors:** Max Kailler Smith, Marcia Grabowecky, Satoru Suzuki

**Affiliations:** ^1^Department of Psychology, Northwestern University, Evanston, Illinois 60208; ^2^Department of Psychology and Interdepartmental Neuroscience, Northwestern University, Evanston, Illinois 60208

**Keywords:** dynamics, EEG, neural oscillation, peak-alpha frequency, posterior–anterior gradient

## Abstract

Peak-alpha frequency varies across individuals and mental states, but it also forms a negative gradient from posterior to anterior regions in association with increases in cortical thickness and connectivity, reflecting a cortical hierarchy in temporal integration. Tracking the spatial standard deviation of peak-alpha frequency in scalp EEG, we observed that a posterior-to-anterior gradient dynamically formed and dissolved. Periods of high spatial standard deviation yielded robustly negative posterior-to-anterior gradients—the “gradient state”—while periods of low spatial standard deviation yielded globally converged peak-alpha frequency—the “uniform state.” The state variations were characterized by a combination of slow (0.3–0.5 Hz) oscillations and random-walk-like fluctuations. They were relatively independently correlated with peak-alpha frequency variations in anterior regions and peak-alpha power variations in central regions driven by posterior regions (together accounting for ∼50% of the state variations), suggesting that two distinct mechanisms modulate the state variations: an anterior mechanism that directly adjusts peak-alpha frequencies and a posterior–central mechanism that indirectly adjusts them by influencing synchronization. The state variations likely reflect general operations as their spatiotemporal characteristics remained unchanged while participants engaged in a variety of tasks (breath focus, vigilance, working memory, mental arithmetic, and generative thinking) with their eyes closed or watched a silent nature video. The ongoing state variations may dynamically balance two global processing modes, one that facilitates greater temporal integration (and potentially also information influx) toward anterior regions in the gradient state and the other that facilitates flexible global communication (via phase locking) in the uniform state.

## Significance Statement

Alpha-band oscillations (8–12 Hz) are implicated in a variety of sensory, attentional, and cognitive processes. Our EEG study reveals that the spatial pattern of alpha-oscillation frequency dynamically varies between forming a negative posterior-to-anterior gradient (i.e., the “gradient state”) and converging globally (i.e., the “uniform state”). The gradient state may facilitate temporal integration (and potentially also information influx) toward anterior regions, whereas the uniform state may promote flexible global communication via phase locking. Our results further suggest that the variations between the gradient and uniform states are controlled by two distinct mechanisms: an anterior mechanism that directly adjusts alpha-oscillation frequencies and a posterior mechanism that indirectly adjusts them by increasing or decreasing global oscillatory entrainment in the upper alpha band.

## Introduction

Alpha-band oscillations are the most salient and ubiquitous features of oscillatory neural activity detected in noninvasive recordings of electrophysiological activity (e.g., EEG, MEG) in humans. Accordingly, peak-alpha frequency, i.e., the frequency at which oscillatory power is maximum within the alpha range, has long been a focus of human electrophysiological research. Numerous studies have demonstrated that peak-alpha frequency stably differs across individuals, increases in childhood but decreases after ∼20 years of age, tends to increase with task demands and arousal but decreases with continued task engagement, varies in relation to emotional valence, and influences sensory processing (see Mierau et al., 2017 for a review; see Scally et al., 2018; Benwell et al., 2019; Nelli et al., 2021; Trajkovic et al., 2023, 2024 for some recent results).

While a large body of research has elucidated trait, state, and modulatory aspects of peak-alpha frequency, recent studies have demonstrated a link between the spatial pattern of peak-alpha frequency and general cortical gradients of neuroanatomical and neurophysiological features. Specifically, a recent MEG study demonstrated that peak-alpha frequency generally forms a negative gradient from posterior to anterior regions, slowing toward anterior regions in association with increases in cortical thickness (Mahjoory et al., 2020). Increases in cortical thickness have been linked to increased connectivity (Huntenburg et al., 2017) as well as an increased ratio of feedback to feedforward connections (Jasmin et al., 2019) which may contribute to longer temporal integration (Salvador et al., 2005; Baria et al., 2013; Murray et al., 2014; Baldassano et al., 2017; Huntenburg et al., 2018). These associations suggest that the negative posterior-to-anterior gradient of peak-alpha frequency reflects the cortical hierarchy in temporal integration (Chaudhuri et al., 2015; Mahjoory et al., 2020). Further, the gradient may facilitate information flow from posterior to anterior regions given that the negative posterior-to-anterior gradient may be accompanied by traveling waves that follow the gradient (Ermentrout and Kleinfeld, 2001; Zhang et al., 2018).

In the current study, we consistently replicated the negative posterior-to-anterior gradient of peak-alpha frequency in scalp EEG while participants engaged in a variety of behavioral tasks including breath focus, vigilance, working memory, mental arithmetic, and generative thinking with their eyes closed (usually generating minimal muscle artifacts) and also while they viewed a silent nature video. Interestingly, we observed that the gradient dynamically formed and dissolved. Tracking the spatial variability (quantified by the spatial standard deviation) of peak-alpha frequency with reasonable temporal resolution (370 ms wavelet standard deviation; see Materials and Methods) and spectral resolution (∼1 Hz), we observed that the spatial pattern of peak-alpha frequency varied between a “uniform state” where peak-alpha frequencies globally converged at ∼11 Hz and a “gradient state” where they gradually decreased from ∼11 Hz in posterior regions to ∼8 Hz in anterior regions. These variations demonstrate that while the negative posterior-to-anterior gradient (potentially reflective of the cortical hierarchy in temporal integration) is consistently observed in time-averaged EEG and MEG activity, there are also mechanisms that dynamically form and dissolve the gradient (anticipated by Chaudhuri et al., 2015; Mahjoory et al., 2020).

The goal of the current study was to identify the processes that drive these state variations. By applying various spatial filters (including those derived from principal components), we discovered that the state variations are independently associated with (1) coordinated variations in peak-alpha frequency in anterior regions and (2) coordinated variations in peak-alpha power in central regions driven by posterior regions, with the two variables accounting for ∼50% of the state variations. Overall, our results suggest that the spatial pattern of peak-alpha frequency varies between the gradient state, facilitating temporal integration (and potentially also information influx) toward anterior regions, and the uniform state, facilitating flexible global communication via phase locking. Our results further suggest that these state variations, characterized by a combination of slow (0.3–0.5 Hz) oscillations and random-walk-like fluctuations, may be controlled by two distinct mechanisms, an anterior mechanism that directly adjusts peak-alpha frequencies and a posterior mechanism that indirectly adjusts them by influencing synchronization.

## Materials and Methods

### Participants and behavioral tasks

Thirty Northwestern University students and individuals from the Evanston/Chicagoland community (20 women; ages 18–39 years, *M *= 23.9, *SD *= 5.3) participated. They were tested individually in a dimly lit room. The study was approved by the Northwestern University Institutional Review Board, and each participant provided a written consent. Data from one participant were excluded from analyses because they unpredictably burst into laughter during the experiment, so our sample size was 29. Each participant performed five behavioral tasks in a fixed order with their eyes closed with pink noise playing through speakers located on a table in front of the participant. Each task was performed continuously for 3 min. In the breath focus task, participants were instructed to focus on the most salient physical sensations of their breath while maintaining natural breathing. In the vigilance task, participants were instructed to listen to background pink noise to detect 15 instances (whose intervals were pseudo-randomly varied between 4 and 20 s) of a brief (200 ms) 15% volume decrement, pressing a button upon detection of each volume decrement. In the 2-back task, an auditory letter was presented every 2 s, and participants were instructed to count the number of instances of a letter being the same as the one presented two letters prior. In the countdown-by-7 task, participants were instructed to count down from a given four-digit number by sevens and to report the final number at the end of the 3 min period. In the alternative-use task, participants were instructed to think about unusual uses of a common object (e.g., shoe) throughout the 3 min period and to report what they thought was the most unusual use at the end. These tasks are not described in greater detail as the purpose of including them was to demonstrate the task independence (generalizability) of our EEG results. Importantly, we chose a set of tasks that engaged a variety of attention and cognitive processes including focusing on body sensations, sustained attention, working memory, mental arithmetic, and generative thinking.

In response to reviewers asking whether our results would generalize to when the eyes were open, we additionally analyzed a 3 min segment (per participant) of our prior EEG data recorded while participants viewed a silent nature video. This nature video condition was included to see if our EEG results involving posterior alpha oscillations, which are prominent when the eyes are closed, generalized to natural viewing. Twenty-one Northwestern University students (13 women and 1 nonbinary person; ages 18–22 years, *M *= 20.0, *SD *= 1.2) participated. The study was approved by the Northwestern University Institutional Review Board, each participant provided written consent, and they were tested individually in a dimly lit room. A generic nature video was presented on a 13 in., 2017 MacBook Pro equipped with 2880(H)-by-1800(V)-pixel-resolution LCD display with normal brightness and contrast settings, placed 100 cm in front of participants, subtending ∼16°(H)-by-10°(V) of visual angle. The EEG data from the nature video condition were previously analyzed for different purposes (Menceloglu et al., 2021a,b, 2024).

### EEG recording and preprocessing

While participants engaged in the breath focus, vigilance, 2-back, countdown-by-7, and alternative-use tasks with their eyes closed or while a separate group of participants viewed a silent nature video, EEG was recorded from 64 scalp electrodes (signals from noise-prone electrodes, Fpz, Iz, T9, and T10, were excluded from analyses) at a sampling rate of 512 Hz using a BioSemi ActiveTwo system (see www.biosemi.com for details). Electrooculographic activity was monitored using four face electrodes, one placed lateral to each eye and one placed beneath each eye. Two additional reference electrodes were placed on the left and right mastoids. The EEG data were preprocessed using EEGLAB and ERPLAB toolboxes for MATLAB (Delorme and Makeig, 2004; Lopez-Calderon and Luck, 2014). Data were rereferenced off-line to the average of the two mastoid electrodes. For the 29 participants who performed the various behavioral tasks with their eyes closed, EEG signals were bandpass-filtered at 0.01–80 Hz and notch-filtered at 60 Hz (to remove power-line noise that affected the EEG signals from some participants). For the 21 participants who viewed a silent nature video, EEG signals were high-pass-filtered at 0.01 Hz without notch filtering at 60 Hz. Note that the removal of the 60 Hz line noise was inconsequential for the current study because we focused on alpha-band oscillations. For the eyes-closed conditions, we visually inspected the EEG waveforms (after applying the surface-Laplacian transform and taking the temporal derivative; see below) as well as the time series of peak-alpha power from all sites per condition per participant and removed intervals that appeared to contain EEG artifacts that affected peak-alpha power. The cleanup resulted in the removal of 0.6% of the data on average as well as the interpolation of one noisy electrode, POz, by replacing its signals with those averaged from its neighbors, Pz, PO3, PO4, and Oz, for one participant in the countdown-by-7 condition. We note that the pattern of results was virtually identical with or without removing these suspected EEG artifacts. Although the EEG data from the nature video condition contained blinks and eye movements, we did not remove them as doing so (e.g., using ICA) may distort oscillatory EEG signals. Nevertheless, the results obtained with the cleaned EEG data from the eyes-closed conditions generalized to the nature video condition, indicating that the analyses presented here are resistant to typical EEG artifacts.

### Estimating dura sources by surface-Laplacian-transforming EEG signals

EEG source reconstruction methods constrained by structural MRI and fMRI localizers obtained from each participant may achieve superior source reconstruction with models customized for each participant (Cottereau et al., 2015). Such an approach, however, was unavailable to us (and for many EEG studies) as we had neither structural MRI nor fMRI data for our participants. Among the noncustomized source-imaging methods, we chose the surface-Laplacian transform that (theoretically) estimates the spatial distribution of macroscopic current sources/sinks on the dura surface. The surface-Laplacian transform has been shown to produce similar dura sources to those inferred by deconvolving scalp EEG potentials using a generic model of thicknesses and impedances of the scalp and skull (Nunez et al., 1994). Popular source-imaging methods such as sLORETA and Beamforming have been shown to approximate simulated sources and/or to extract neural correlates of behaviors to a similar degree as the surface-Laplacian transform (Tenke and Kayser, 2012; Cohen, 2015). Further, there is no evidence (to our knowledge) to suggest that these popular source-imaging methods provide greater spatial resolution than the surface-Laplacian transform. Thus, our preference was to use the latter because it is the most general source-imaging method that is least reliant on model-specific assumptions and free parameters (Hjorth, 1980; Nunez et al., 1994; Kayser and Tenke, 2006; Nunez and Srinivasan, 2006; Tenke and Kayser, 2012).

The surface-Laplacian transform is expected to reduce volume-conduction effects from substantially greater than 5 cm in raw EEG to within 1–3 cm (Nunez et al., 1994; Tenke and Kayser, 2012; Cohen, 2014; Menceloglu et al., 2024) which approximately corresponds to the average spacing of electrodes in our 64-channel montage. For our implementation of the surface-Laplacian transform, we used Perrin's and colleague's algorithm (Perrin et al., 1987, 1989a,b) with a “smoothness” value, $\lambda \;=\;10^{-5}$ (as recommended for 64 channels; Cohen, 2014). We refer to the surface-Laplacian-transformed EEG signals that represent the macroscopic current sources/sinks on the dura surface under the 60 scalp sites (with the four noise-prone sites excluded from analyses) simply as EEG signals. These EEG-recording and preprocessing procedures were similar to those used in our prior studies (Menceloglu et al., 2021a,b, 2024).

### EEG analysis

#### Taking the temporal derivative

**Figure 1.**
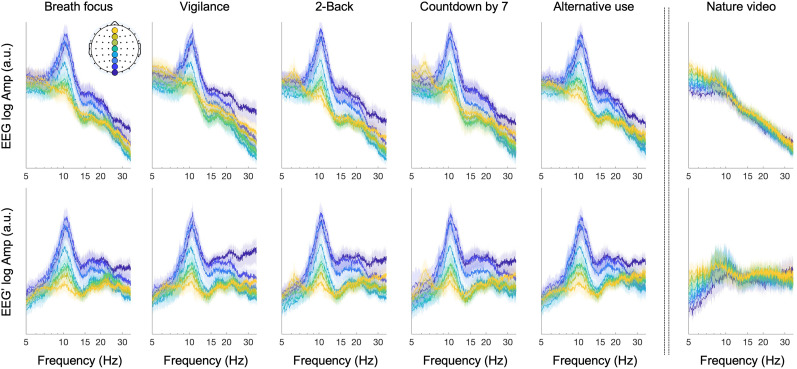
Taking the temporal derivative of EEG (EEG′) flattens FFT amplitude spectra (the data for midline sites shown as a representative example). FFTs were computed for nonoverlapping 20 s time segments and averaged (to obtain a smoother profile) for each of the eight midline sites (Oz, POz, Pz, CPz, Cz, FCz, Fz, and AFz; color coded from blue to yellow) per participant. The columns correspond to the six behavioral conditions (participants closed their eyes in all but the nature video condition). The top row shows the FFTs based on EEG, whereas the bottom row shows FFTs based on the temporal derivative of EEG (EEG′). These FFTs were computed before splicing out the intervals suspected of artifacts/noise (see Materials and Methods), so the data from two participants (one in the vigilance condition and the other in the countdown-by-7 condition) were excluded as their data contained multiple intervals of artifacts/noise. Note that taking the temporal derivative of EEG (EEG′) generally flattened the $1/f^{\beta }$ slope, especially at higher frequencies. The shaded regions represent the 95% confidence intervals.

We needed to track the peak-alpha frequency and power at each site at a subsecond timescale and with reasonable spectral resolution. The general $1/f^{\beta }$ spectral background in EEG may interfere with the identification of the frequencies and powers at spectral maxima. A commonly employed strategy to circumvent this problem is to compute FFTs over partially overlapping time windows of several seconds or longer and then fit a power function to each time-windowed FFT to remove the $1/f^{\beta }$ component (Chiang et al., 2011; van Albada and Robinson, 2013; Donoghue et al., 2020). However, it is difficult to reliably estimate the $1/f^{\beta }$ component on a subsecond timescale. Note that β in the $1/f^{\beta }$ spectral background varies around 1 (see He, 2014 for a review of the various factors that influence β, and Gao et al., 2017 for contributions of the excitatory and inhibitory dynamics to β). Although taking the temporal derivative of EEG ( (ΔEEG/Δt), where Δt is the temporal resolution, i.e., 1/512 s) would completely neutralize the $1/f^{\beta }$ component only when β=1, the method worked well in our prior studies (Menceloglu et al., 2021a, b, 2024). Note that any time series, f(t), can be expressed as an integral over its sinusoidal components, $f(t)=(1/\sqrt{2\pi })\int_{-\infty }^{\infty }g(\omega )e^{i\omega t}d\omega$ with $g(\omega )=(1/\sqrt{2\pi })\int_{-\infty }^{\infty }\;f(t)e^{i\omega t}dt$, where ω/2π is the frequency (Fourier's theorem). Because taking the temporal derivative merely multiplies each sinusoidal component by its frequency (from the chain rule of differentiation), taking the temporal derivative adds the log–log slope of 1 to an amplitude spectrum to cancel the log–log slope of the aperiodic component if β=1. If β is different than 1, taking the temporal derivative will either under- or overcompensate the negative aperiodic slope. For the EEG data analyzed here, taking the temporal derivative clearly reduced/removed the negative slopes of the aperiodic components from the amplitude spectra (Fig. 1).

#### Time–frequency decomposition using Morlet wavelets

To track EEG power spectra at high temporal and spectral resolution, we used a Morlet wavelet-convolution method suitable for time–frequency decomposition of signals containing multiple oscillatory sources of different frequencies (see Cohen, 2014 for a review of different methods for time–frequency decomposition). A Morlet wavelet is a Gaussian-windowed complex sinusoidal template characterized by its frequency as well as its temporal and spectral widths that limit its temporal and spectral resolution, respectively. We convolved each EEG waveform (i.e., its temporal derivative) with a set of wavelets tuned to a range of frequencies, yielding a time series of complex values per wavelet frequency. The power and phase of each extracted sinusoidal component at each timepoint were given by the modulus squared (power) and the arc tangent of the ratio of the imaginary to the real component (phase). We used a set of wavelets with 160 frequencies, $f_{w}$'s, ranging from 5 to 15 Hz (as the primary alpha-oscillation frequency may vary from 6 to 13 Hz across individuals; Chapeton et al., 2019). The $f_{w}$'s were logarithmically spaced as neural temporal-frequency tunings tend to be approximately logarithmically scaled (Hess and Snowden, 1992; Liu et al., 2007). The accompanying *n* factor (roughly the number of cycles per wavelet, with the precise definition, n=2πf⋅SD, where SD is the wavelet standard deviation) was also logarithmically spaced between 11.7 and 35. This spacing yielded a temporal resolution of SD* *= 370 ms and a spectral resolution of FWHM (full width at half maximum of wavelet spectrum) = 1.0 Hz, which were virtually invariant across the range of wavelet frequencies.

We thus obtained the power spectrum in the 5–15 Hz range as a function of time with a temporal resolution of 370 m (wavelet standard deviation) and the spectral resolution of 1.0 Hz (FWHM). To track peak-alpha frequency and power, in each power spectrum (per timepoint), we identified the curvature maximum that had the highest power and then registered the corresponding frequency and power as the peak-alpha frequency and peak-alpha power. Although we could have simply identified the spectral peak with the highest power, we observed that curvature maxima identified oscillatory frequencies with greater sensitivity and precision than spectral peaks (Suzuki et al., 2023).

## Results

**Figure 2.**
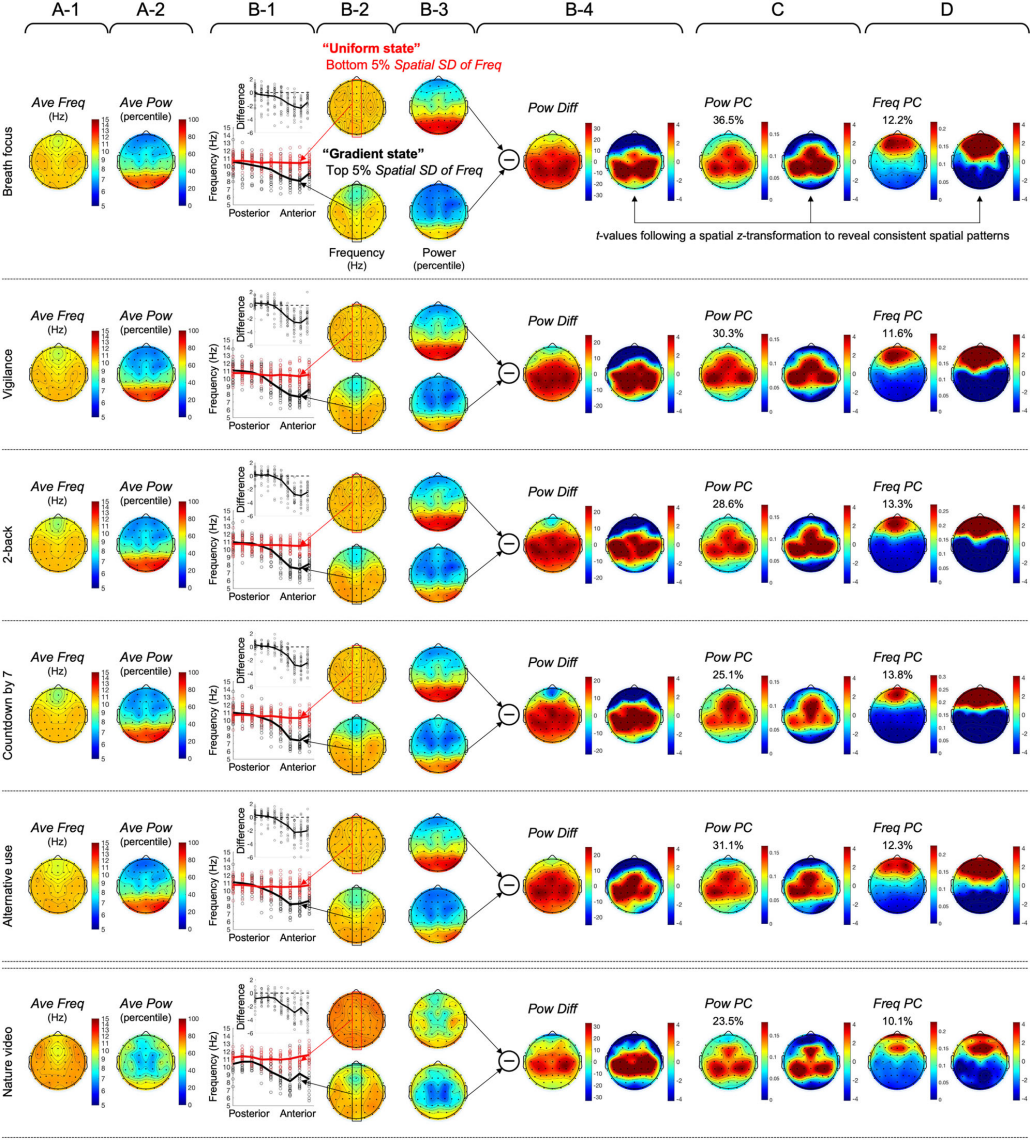
Various spatial maps (columns) of peak-alpha frequency and power for the six behavioral conditions (rows). ***A***, The spatial pattern of time-averaged peak-alpha frequency (Hz), *Ave Freq* (***A*-1**), and the spatial pattern of time-averaged peak-alpha power (percentile), *Ave Pow* (***A*-2**). Note that peak-alpha frequency diminished from posterior to anterior regions along the midline (***A*-1**), whereas peak-alpha power was elevated in posterior regions in the eyes-closed conditions (***A*-2**, top five rows). ***B***, The spatial patterns of peak-alpha frequency (Hz) averaged during the periods of low (bottom 5%; ***B*-2**, upper) and high (top 5%, ***B-*****2**, lower) spatial standard deviation, with the values along the midline axis plotted in ***B*-1** (the circles representing individual participants) with the top inset showing the difference between the high- and low-spatial-standard-deviation states. Note that peak-alpha frequency globally converged at ∼11 Hz in the low-spatial-standard-deviation state, the “uniform state” (***B*-2**, upper, and ***B*-1**, red curve), whereas a robust negative posterior-to-anterior frequency gradient emerged in the high-spatial-standard-deviation state, the “gradient state” (***B*-2**, lower, and ***B*-1**, black curve). When the spatial patterns of peak-alpha frequency were examined for intermediate levels of spatial standard deviation, the posterior-to-anterior gradient monotonically increased as spatial standard deviation increased (Extended Data Fig. 2-1). ***B*-3** shows the spatial patterns of peak-alpha power (percentile) averaged for the periods of low and high spatial standard deviation of peak-alpha frequency, with their difference, *Pow Diff*, shown in ***B*-4**, accompanied by the right plot showing *t*-values (following a spatial *z*-transformation) indicating the statistically reliable spatial pattern (|*t*| > 3.75 indicating Bonferroni-corrected statistical significance at *p *= 0.05, 2-tailed). Note that peak-alpha power was broadly elevated in central regions in the uniform state relative to the gradient state (***B*-4**). ***C***, The spatial loading of the first principal component of peak-alpha power, *Pow PC*, with the right plot showing *t*-values (following a spatial *z*-transformation) indicating the statistically reliable spatial pattern (|*t*| > 3.75 indicating Bonferroni-corrected statistical significance at *p *= 0.05, 2-tailed). ***D***, The spatial loading of the first principal component of peak-alpha frequency, *Freq PC*, with the right plot showing *t*-values (following spatial *z*-transformation) indicating the statistically reliable spatial pattern (|*t*| > 3.75 indicating Bonferroni-corrected statistical significance at *p *= 0.05, 2- tailed). The percentage values shown in ***C*** and ***D*** indicate the percentages of the variances in peak-alpha power and frequency accounted for by the corresponding first principal components. Note that *Pow PC* is centrally localized similar to *Pow Diff*, whereas *Freq PC* is anteriorly localized.

We tracked peak-alpha frequency and power at each site with 370 ms temporal resolution (wavelet temporal standard deviation) and 1.0 Hz spectral resolution (FWHM). In our time-averaged data, we replicated the negative posterior-to-anterior gradient of peak-alpha frequency previously reported with MEG (Mahjoory et al., 2020) using EEG in all six behavioral conditions. Peak-alpha frequency decreased from ∼11 Hz in posterior regions to ∼9 Hz in anterior regions along the midline (Fig. 2*A*-1). We also replicated the typical finding of elevated peak-alpha power in posterior regions when the eyes are closed (Fig. 2*A*-2, top five rows) relative to when the eyes are open (Fig. 2*A*-2, bottom row). Note that all means and standard deviations of peak-alpha frequency were computed in log as our wavelet frequencies were logarithmically spaced (see Materials and Methods).

**Figure 3.**
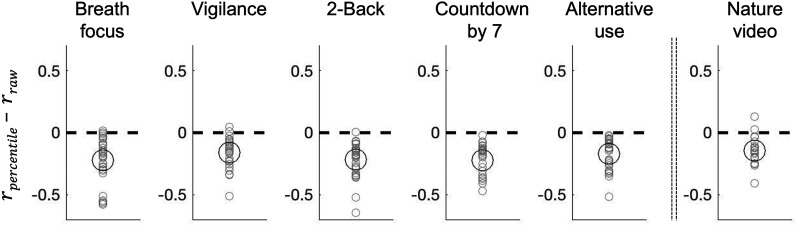
Correlation between the spatial standard deviation of peak-alpha frequency and peak-alpha power in the central regions [corresponding to *Pow Diff* (Fig. 2*B*-4, left)] computed with percentile versus raw power. For each participant in each behavioral condition, we computed the difference in Pearson's *r* (Fisher *z* transformed) between the value computed with percentile power and that computed with raw power, $r_{\mathrm{percentile}}-r_{\mathrm{raw}}$. Both correlations were negative (not shown here) as implied by the fact that peak-alpha power was elevated in the central regions when the spatial standard deviation of peak-alpha frequency was low relative to when it was high (Fig. 2*B*-4). Note that the negative correlation was consistently stronger using percentile power than using raw power for nearly all participants (gray circles) in all behavioral conditions. The large black circles represent the means.

10.1523/ENEURO.0273-24.2024.f2-1 Figure 2-1 Spatial patterns of peak-alpha frequency (left columns) and peak-alpha power (right columns) at different percentile levels of the spatial standard deviation of peak-alpha frequency (*Spatial SD of Freq*) for the six behavioral conditions (column pairs). The spatial patterns are shown for ten evenly spaced 5% intervals of *Spatial SD of Freq.* The lowest and the highest levels correspond to the “uniform” and “gradient” states, respectively, shown in Figure 2B-2 and 2B-3. Note that as *Spatial SD of Freq* increased, the negative posterior-to-anterior gradient of peak-alpha frequency gradually emerged and strengthened without going through any distinct intermediate patterns. This indicates that *Spatial SD of Freq* (spatial standard deviation of peak-alpha frequency) provides an appropriate measure to quantify and track the variations between the uniform and gradient states. Download Figure 2-1, TIF file.

### The spatial pattern of peak-alpha frequency varies between the “uniform” and “gradient” states

The goal of the current study was to gain insights into how the posterior-to-anterior gradient of peak-alpha frequency dynamically formed and dissolved. We tracked the spatial standard deviation of peak-alpha frequency, *Spatial SD of Freq*, and examined the spatial patterns of peak-alpha frequency and power when *Spatial SD of Freq* was minimal (bottom 5%) and when it was maximal (top 5%). It is clear that the instances of low *Spatial SD of Freq* corresponded to spatially homogeneous peak-alpha frequency—the “uniform” state (Fig. 2*B*-2, upper)—whereas the instances of high *Spatial SD of Freq* corresponded to an enhanced posterior-to-anterior gradient—the “gradient” state (Fig. 2*B*-2, lower). In all behavioral conditions, peak-alpha frequency converged at ∼11 Hz along the midline axis in the uniform state (Fig. 2*B*-1, red curve), whereas it decreased from ∼11 Hz (posterior) to 7–8 Hz (anterior) in the gradient state (Fig. 2*B*-1, black curve). The circles in Figure 2*B*-1 represent the data from individual participants, and the upper inset shows the difference (the gradient state minus the uniform state). When the spatial patterns of peak-alpha frequency were examined for intermediate levels of *Spatial SD of Freq*, the posterior-to-anterior gradient monotonically increased as *Spatial SD of Freq* increased (Extended Data Fig. 2-1). Thus, *Spatial SD of Freq* (the spatial standard deviation of peak-alpha frequency) provides an appropriate measure for tracking the variations between the uniform and gradient states.

The results so far demonstrate that (1) the spatial organization of peak-alpha frequency varies between the uniform and gradient states and (2) the spatial standard deviation of peak-alpha frequency, *Spatial SD of Freq*, can be used to track the dynamic variations between the uniform and gradient states. We conducted a series of analyses to elucidate the processes that may drive the state variations.

### The variations between the uniform and gradient states are correlated with peak-alpha power variations in central regions

**Figure 4.**
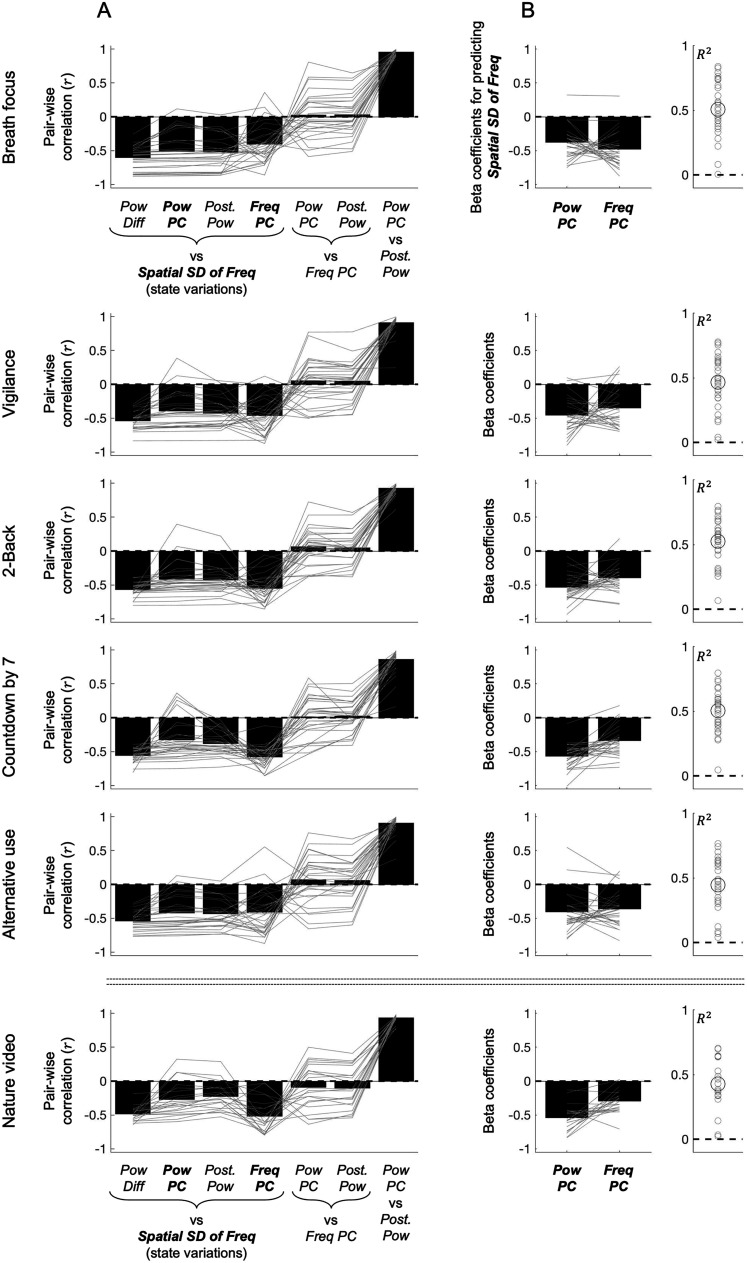
Temporal correlations between the spatial standard deviation of peak-alpha frequency (*Spatial SD of Freq*, our measure of the state variations between the uniform and gradient states) and various spatial components of peak-alpha power and frequency for the six behavioral conditions (rows). ***A***, Correlations (Pearson's *r*) between *Spatial SD of Freq* (the state variations) and *Pow Diff* (the centrally localized variations in peak-alpha power weighted by the spatial pattern of power difference between the uniform and gradient states; Fig. 2*B*-4), *Pow PC* (the centrally localized variations in the first principal component of peak-alpha power; Fig. 2*C*), *Post. Pow* (the posteriorly localized variations in peak-alpha power weighted by the spatial pattern of average power in the eyes-closed conditions; Fig. 2*A*-2, top five rows), and *Freq PC* (the anteriorly localized variations in the first principal component of peak-alpha frequency; Fig. 2*D*), as well as correlations between *Freq PC* and *Pow PC*, *Freq PC* and *Post. Pow*, and *Pow PC* and *Post. Pow* (from left to right). For the nature video condition (bottom row), because the average peak-alpha power was no longer posteriorly focused (Fig. 2*A*-2, bottom row) due to the eyes being open, *Post. Pow* was computed by weighting peak-alpha power by the posteriorly localized spatial pattern averaged from the five eyes-closed conditions. The gray lines represent individual participants and the black bars represent the means. The near parallel lines across the first three bars (in all conditions) indicate that *Pow Diff*, *Pow PC*, and *Post. Pow* were equivalently negatively correlated with *Spatial SD of Freq* (the state variations) except for a few participants. While the fourth bar was also reliably negative, the crossing of the gray lines suggest that *Freq PC* was negatively associated with *Spatial SD of Freq* in a different way than *Pow Diff*, *Pow PC*, and *Post. Pow* were. Accordingly, the fifth and sixth bars indicate that there were no consistent correlations between *Freq PC* and *Pow PC* or between *Freq PC* and *Post. Pow*, though the individual differences were large (see main text). The tall rightmost bar indicates that *Pow PC* and *Post. Pow* were highly correlated with each other. The overall pattern suggests that *Pow PC* and *Freq PC* account for nonoverlapping dynamics of the variations in *Spatial SD of Freq* (the state variations). ***B***, Beta coefficients for predicting *Spatial SD of Freq* with *Pow PC* and *Freq PC*. The gray lines represent individual participants and the black bars represent the means. The proportion of the variance of *Spatial SD of Freq* (the state variations) accounted for jointly by *Pow PC* and *Freq PC* is shown as *R*^2^ on the right with gray circles representing individual participants and the large black circle representing the mean. Note that nearly all participants yielded negative beta coefficients for both *Pow PC* and *Freq PC* with the pair accounting for a substantial proportion (∼50% on average) of the state variations.

Interestingly, peak-alpha power was consistently elevated in the uniform state relative to the gradient state (Fig. 2*B*-3, upper vs lower). Taking the difference, *Pow Diff*, showed that peak-alpha power was broadly elevated in central regions in the uniform state in all behavioral conditions (Fig. 2*B*-4, left). To statistically evaluate the central localization of the power elevation (in the uniform state), we plotted *t*-values after spatially *z*-transforming power values per participant (to normalize the spatial pattern per participant; Fig. 2*B*-4, right). Given that |*t*| = 3.34 corresponds to Bonferroni-corrected statistical significance at *α *= 0.05 (two-tailed), the analysis confirmed that peak-alpha power was broadly elevated in central regions in the uniform state relative to the gradient state.

Note that we analyzed peak-alpha power in percentile. As spectral power in human EEG tends to be approximately exponentially distributed (Freyer et al., 2009; Menceloglu et al., 2021a), the percentile transformation emphasized power variations in the low to middle ranges. We used percentile-transformed power because the variations in peak-alpha power were more strongly correlated with the state variations (i.e., with *Spatial SD of Freq*) when we used percentile (rather than raw) power. Specifically, we derived a univariate time series of peak-alpha power variations most likely associated with the state variations by spatially averaging peak-alpha power (at each timepoint) while weighting the power from each scalp site according to the centrally localized spatial pattern of the power difference between the uniform and gradient states, *Pow Diff* (Fig. 2*B*-4, left, but computed separately for each participant). As expected, this *Pow Diff* time series was negatively correlated with the state variations for all participants in all behavioral conditions (Fig. 4*A*, the leftmost bar, *Pow Diff* vs *Spatial SD of Freq*). The correlation computed with percentile power values was consistently stronger than that computed with raw power values, indicated by the difference, $r_{\mathrm{percentile}}-r_{\mathrm{raw}}$ (Fisher *z* transformed), being consistently negative for nearly all participants in all behavioral conditions (Fig. 3). This suggests that the state variations between the uniform and gradient states are primarily associated with peak-alpha power variations in the low to middle range (emphasized by the percentile transformation) in central regions (Fig. 2*B*-4).

### The centrally localized peak-alpha power variations associated with the state variations reflect dominant power variations driven by posterior regions

To gain insights into the sources of the centrally localized peak-alpha power variations associated with the state variations, we examined (1) spatiotemporally dominant variations in peak-alpha power and (2) peak-alpha power variations in the posterior regions where the average power was maximal (when the eyes were closed). Spatiotemporally dominant peak-alpha power variations were computed as the first principal component of peak-alpha power (*Pow PC*), which accounted for 23.5–36.5% of the variance across the six behavioral conditions. Note that the centrally localized spatial loading (Fig. 2*C*) of *Pow PC* generally overlapped the centrally localized peak-alpha power variations associated with the state variations (*Pow Diff*; Fig. 2*B*-4). Further, *Pow PC* was similarly correlated with the state variations (*Spatial SD of Freq*) as *Pow Diff*, indicated by the generally parallel gray lines connecting the first two bars in Figure 4*A* (except for a few participants). These results suggest that the state variations are primarily associated with the spatiotemporally dominant peak-alpha power variations captured by the first principal component.

The peak-alpha power variations in the posterior regions where the average power was maximal (when the eyes were closed) were obtained by spatially averaging peak-alpha power (at each timepoint) while weighting the power from each scalp site according to the spatial pattern of the average power (*Ave Pow*, Fig. 2*A*-2, but computed separately for each participant). Because the average power was posteriorly localized, we call this time series, *Post. Pow*. As the average peak-alpha power was not posteriorly localized in the nature video condition (due to the eyes being open, Fig. 2*A*-2, bottom row), the peak-alpha power in this condition was weighted by the average spatial pattern from the five eyes-closed conditions. The posteriorly localized *Post. Pow* and centrally localized *Pow PC* were equivalently negatively correlated with the state variations (*Spatial SD of Freq*) as indicated by the nearly parallel gray lines between the second and third bars in Figure 4*A* and were highly correlated with each other (the rightmost bar in Fig. 4*A*).

**Figure 5.**
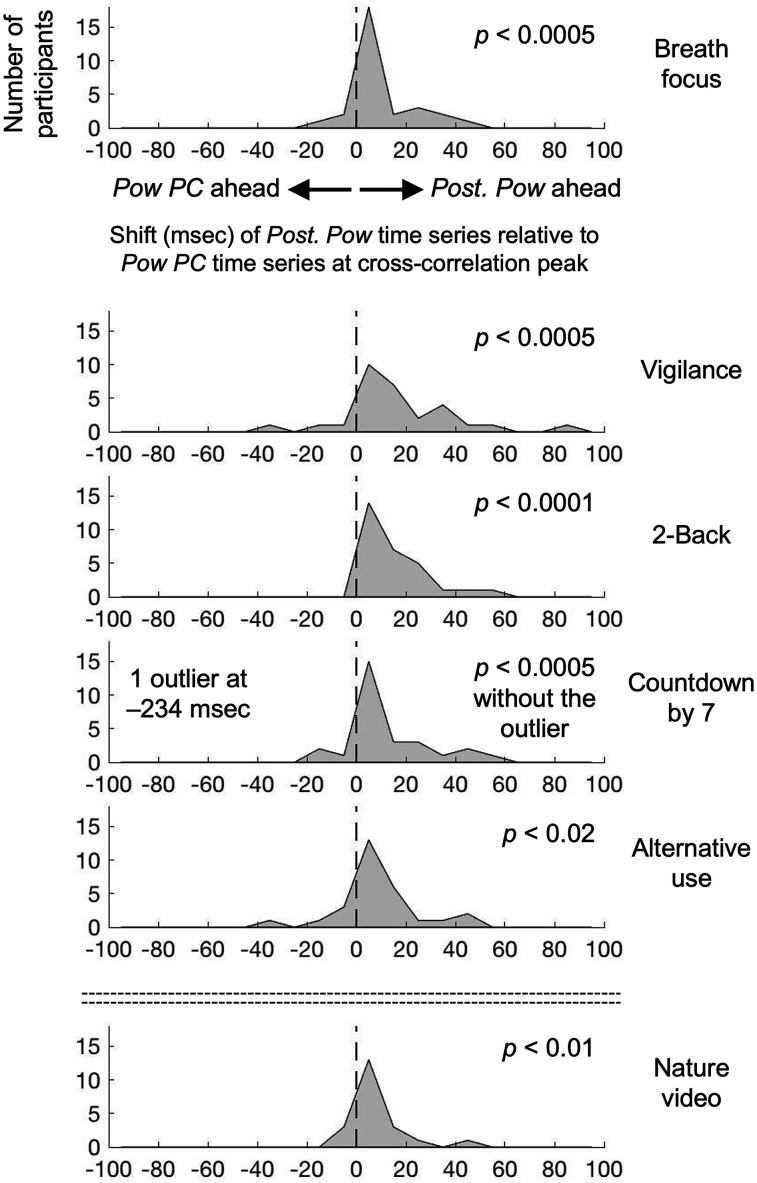
Distribution (across participants) of the time shift (msec) of the posteriorly localized *Post. Pow* time series relative to the centrally localized *Pow PC* time series at their cross-correlation peak, for the six behavioral conditions (rows). Note that the distribution is positively shifted in all behavioral conditions, indicating that the posteriorly localized *Post. Pow* varied ahead of the centrally localized *Pow PC*.

Given that the central (*Pow PC*) and posterior (*Post. Pow*) variations in peak-alpha power were equivalently associated with the state variations, we examined the cross correlation between *Pow PC* and *Post. Pow* to see whether one region tended to drive the other region. For each participant (and each behavioral condition), we computed the time shift of *Post. Pow* relative to *Pow PC* at their cross-correlation peak. A negative value indicates that *Pow PC* varied ahead of *Post. Pow*, whereas a positive value indicates that *Post. Pow* varied ahead of *Pow PC*. Although there were individual differences, the time shifts were positive for most participants in all behavioral conditions (Fig. 5). This suggests that whether the eyes are closed or open, the peak-alpha power variations in posterior regions (*Post. Pow*) drive the dominant peak-alpha power variations in central regions (*Pow PC*) associated with the state variations.

### The state variations are also associated with anteriorly localized variations in peak-alpha frequency

In addition to the first principal component of peak-alpha power, *Pow PC*, we also examined the first principal component of peak-alpha frequency, *Freq PC*, which accounted for 10.1–13.8% of the variance across the six behavioral conditions. The anteriorly localized spatial loading of *Freq PC* (Fig. 2*D*) generally overlapped the anterior dip in peak-alpha frequency characteristic of the gradient state (see the lower plots in Fig. 2*B*-2).

*Freq PC* was negatively correlated with the state variations (the fourth bar in Fig. 4*A*) to an equivalent degree as *Pow PC*. Note that *Freq PC*'s and *Pow PC*'s negative correlations with the state variations make sense as we quantified the state variations as the variations in the spatial standard deviation of peak-alpha frequency (*Spatial SD of Freq*). Both lower *Freq PC* (reflecting lower peak-alpha frequency in anterior regions) and lower *Pow PC* (reflecting lower peak-alpha power in central regions) were associated with higher *Spatial SD of Freq* (toward the gradient state), whereas both higher *Freq PC* (reflecting higher peak-alpha frequency in anterior regions) and higher *Pow PC* (reflecting higher peak-alpha power in central regions) were associated with lower *Spatial SD of Freq* (toward the uniform state). While both *Freq PC* and *Pow PC* were negatively correlated with the state variations, the individual differences were inconsistent for the two variables [the near parallel gray lines through the first three bars (*Pow Diff*, *Pow PC*, and *Post. Pow*) crossing into the fourth bar (*Freq PC*) in Fig. 4*A*]. Further, there were no consistent correlations between *Pow PC* and *Freq PC* across participants (the fifth bar in Fig. 4*A*). Thus, *Pow PC* and *Freq PC* likely account for different dynamics of the state variations. To confirm this observation, we ran a linear multiple regression model predicting the state variations with *Pow PC* and *Freq PC*.

Most participants in all behavioral conditions yielded negative beta coefficients for both *Pow PC* and *Freq PC* (Fig. 4*B*, left panel). Further, whereas *Pow PC* and *Freq PC* each accounted for ∼25% of the variance (on average) of the state variations (Fig. 4*A*, the second and fourth bars, generally approaching *r* ∼ 0.5), *Pow PC* and *Freq PC* together accounted for ∼50% of the variance (on average) in the multiple regression model (Fig. 4*B*, right panel). These results confirm that alpha-band synchronization spreading from posterior to central regions and alpha-band frequency modulations in anterior regions relatively independently account for the state variations between the uniform and gradient states.

### More positive correlations between *Pow PC* and *Freq PC* predict larger state variations

**Figure 6.**
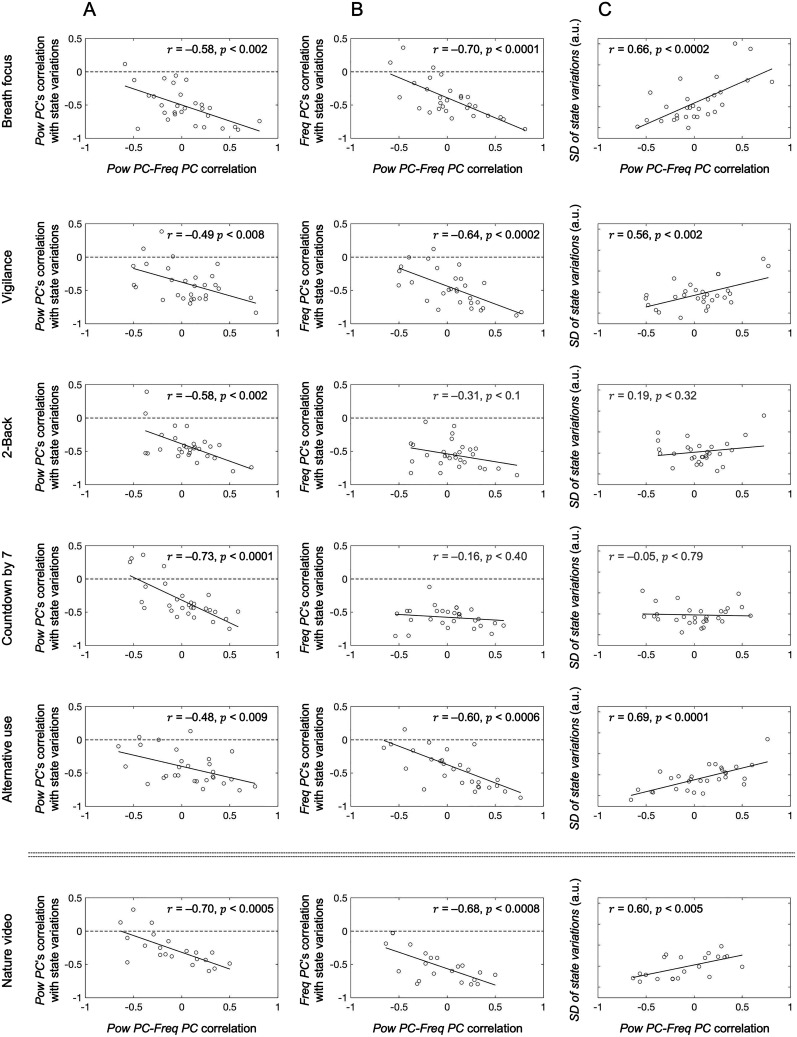
Assessing the impact of the large individual differences in *Pow PC*-*Freq PC* correlation. ***A***, Correlation between *Pow PC*–*Freq PC* correlation and *Pow PC*'s correlation with the state variations (quantified by the spatial standard deviation of peak-alpha frequency). Note that the correlation was negative in all six conditions, indicating that more positive *Pow PC-Freq PC* coupling was associated with stronger negative correlation between *Pow PC* and the state variations. ***B***, Correlation between *Pow PC*–*Freq PC* correlation and *Freq PC*'s correlation with the state variations. Note that the correlation was negative in all conditions except the two conditions that continually engaged working memory (2-back and countdown-by-7), indicating that more positive *Pow PC-Freq PC* coupling was associated with stronger negative correlation between *Freq PC* and the state variations at least when working memory was not continually engaged. ***C***, Correlation between *Pow PC*–*Freq PC* correlation and the magnitude of the state variations (quantified by the standard deviation of the state variations). Note that the correlation was positive in all conditions except the two conditions that continually engaged working memory (2-back and countdown-by-7), indicating that more positive *Pow PC–Freq PC* coupling was associated with larger state variations at least when working memory was not continually engaged.

Although we found no consistent correlations between *Pow PC* and *Freq PC* across participants, the individual differences were large (the gray lines at the fifth bar in Fig. 4*A*). We thus examined the possibility that the correlation between *Pow PC* and *Freq PC* predicted *Pow PC*'s and *Freq PC*'s associations with the state variations. *Pow PC*'s negative correlation with the state variations was greater (i.e., more negative) when *Pow PC* and *Freq PC* were more positively correlated in all six behavioral conditions (Fig. 6*A*). Similarly, *Freq PC*'s negative correlation with the state variations was also greater (i.e., more negative) when *Pow PC* and *Freq PC* were more positively correlated, except in the 2-back and countdown-by-7 conditions (Fig. 6*B*) that required continual engagement of working memory. For these conditions, *Freq PC*'s negative correlation with the state variations was equivalent irrespective of its correlation with *Pow PC* (Fig. 6*B*, flat linear fits in the third and fourth rows). Thus, unless working memory was continually engaged, *Pow PC* and *Freq PC* were more strongly associated with the state variations when they were more positively coupled. One interpretation of this result is that the centrally localized peak-alpha power variations (quantified by *Pow PC*) and the anteriorly localized peak-alpha frequency variations (quantified by *Freq PC*) more strongly drive the state variations when the power and frequency variations are more positively coupled (at least when working memory is not strongly engaged). If so, the state variations should be larger when *Pow PC* and *Freq PC* are more positively correlated (except for the 2-back and countdown-by-7 conditions).

To test this prediction, we quantified the magnitude of the state variations by its temporal standard deviation (SD of state variations), i.e., by the temporal standard deviation of *Spatial SD of Freq* (the spatial standard deviation of peak-alpha frequency). SD of state variations increased when *Pow PC* and *Freq PC* were more positively correlated, except (as expected) for the 2-back and countdown-by-7 conditions (Fig. 6*C*). Thus, stronger positive coupling between alpha-band synchronization in posterior–central regions and alpha-band frequency modulations in anterior regions predicts larger state variations between the uniform and gradient states, except when working memory is continually engaged.

### The state variations comprise slow (0.3–0.5 Hz) oscillations and random-walk-like fluctuations, with dwell times in the uniform and gradient states lasting as long as 1 s

**Figure 7.**
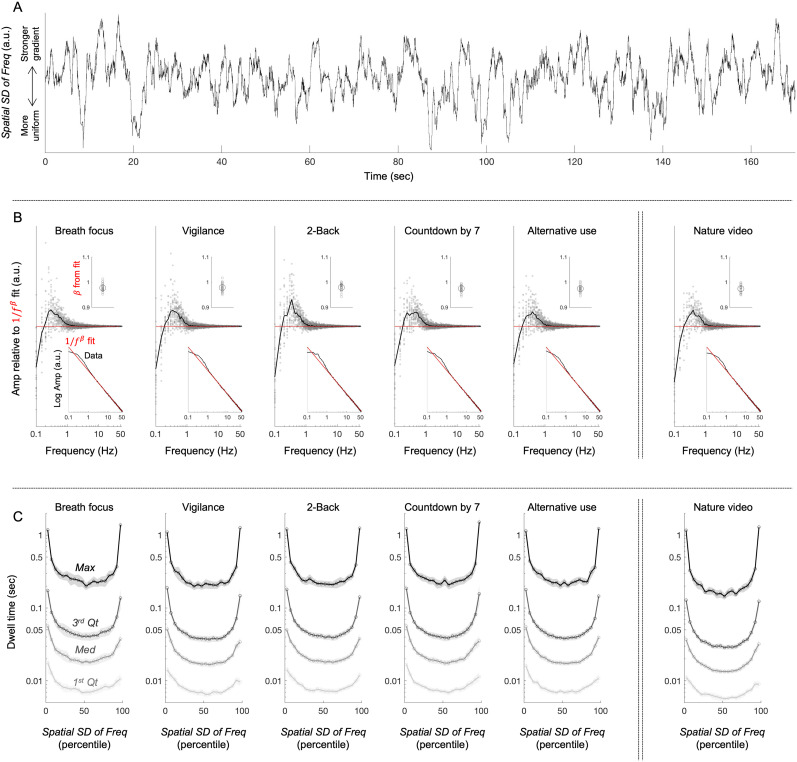
Temporal characteristics of the spatial standard deviation of peak-alpha frequency (*Spatial SD of Freq*, our measure of the state variations between the uniform and gradient states). ***A***, An example of *Spatial SD of Freq* (the state variations) as a function of time for one participant in the vigilance condition. Note that the state variations appear to comprise low-frequency oscillations embedded within a random-walk-like fluctuations. ***B***, FFT amplitude spectrum of the time series of *Spatial SD of Freq*. For each participant, an FFT was computed for each nonoverlapping 20 s interval and averaged (to generate smooth amplitude spectra), and it was fit by a power function, $1/f^{\beta }$ (from 2 to 55 Hz, excluding the low-frequency bump and flattening). The lower inset shows the average FFT (black) and $1/f^{\beta }$ fit (red), with each fit (per condition per participant) exceeding 0.997 in adjusted *r*^2^. The upper inset shows the exponent β of the fit (gray circles representing individual participants and the large black circle representing the mean). The main plot shows the difference between the FFT and the power-function fit (gray circles representing individual participants and the black curve representing the mean). Note that there is a bump (periodicity) at 0.3–0.5 Hz; otherwise, the state variations (*Spatial SD of Freq*) are well described by 1/f fluctuations. ***C***, Dwell times within different percentile ranges of *Spatial SD of Freq*. Each point along the *x*-axis represents a 5% interval, from 0th–5th percentile (which we call the uniform state), 5th–10th percentile, …, 90th–95th percentile, to 95th–100th percentile (which we call the gradient state). Each curve represents a landmark of the dwell-time distribution, the first quartile (1st *Qt*), median (*Med*), third quartile (3rd *Qt*), and maximum (*Max*), each averaged across participants. The shaded gray regions represent the 95% confidence intervals. Note that the dwell times were particularly elevated at the lowest and highest *Spatial SD of Freq* (i.e., in the uniform and gradient states), with the third quartile reaching ∼0.2 s and maximum exceeding 1 s.

In the final set of analyses, we examined the temporal characteristics of the state variations. One can see from an example time series (from one participant in the vigilance condition) that the state variations (*Spatial SD of Freq*) include slow oscillations and random-walk-like fluctuations (Fig. 7*A*). Indeed, the FFT amplitude spectrum of the state variations yielded a bump at 0.3–0.5 Hz, indicative of slow oscillations, but otherwise followed a linear decay in log–log (Fig. 7*B*, lower inset) well fit by $1/f^{\beta }$ with β∼1 (upper inset), indicative of 1/f fluctuations. The $1/f^{\beta }$ component was subtracted from the amplitude spectrum in the main plot in Figure 7*B* to clearly show the low-frequency bump (gray circles showing individual participants’ data). Thus, the state variations comprise slow (0.3–0.5 Hz) oscillations embedded in random-walk-like fluctuations.

The state variations yielded relatively long dwell times at low and high values of *Spatial SD of Freq* (the spatial standard deviation of peak-alpha frequency), corresponding to what we call the uniform and gradient states, respectively. Specifically, we computed dwell times within different ranges (in percentile) of *Spatial SD of Freq*, from 0th–5th percentile (which we call the uniform state), 5th–10th percentile, 10th–15th percentile, …, 90th–95th percentile, to 95th–100th percentile (which we call the gradient state). We examined the first quartile, median, third quartile, and maximum dwell times within each of these percentile ranges. The U-shaped functions (Fig. 7*C*) indicate that the dwell times were symmetrically elevated in the lowest and highest ranges (i.e., in the uniform and gradient states), where the average third quartile dwell times reached 0.2 s and the average maximum dwell times exceeded 1 s.

## Discussion

Prior research has demonstrated a generally negative posterior-to-anterior gradient of peak-alpha frequency, slowing from posterior to anterior regions (Zhang et al., 2018; Mahjoory et al., 2020), that was associated with increases in cortical thickness (Mahjoory et al., 2020). Increases in cortical thickness have been linked to increases in connectivity (Huntenburg et al., 2017) and the ratio of feedback to feedforward connections (Jasmin et al., 2019). Further, regions with increased connectivity tend to show slower fMRI BOLD fluctuations (Salvador et al., 2005; Baria et al., 2013), the timescale of intrinsic fluctuations in spiking activity tends to increase from sensory areas to association areas in the primate cortex (Murray et al., 2014), and the cortical gradient of connectivity is similar to that of temporal integration, generally increasing from posterior to anterior regions (Baldassano et al., 2017; Huntenburg et al., 2018). Taken together, these relations suggest that the negative posterior-to-anterior gradient of peak-alpha frequency reflects the cortical hierarchy in temporal integration (Chaudhuri et al., 2015; Mahjoory et al., 2020).

The posterior-to-anterior gradient may also reflect the information influx into anterior regions given that a primary mechanism of a cortical traveling wave is a linear spatial gradient of oscillation frequency, wherein a wave travels from higher-frequency regions to lower-frequency regions (Ermentrout and Kleinfeld, 2001; Zhang et al., 2018). Nevertheless, although traveling waves (including alpha-band waves) tended to propagate from anterior to posterior when the eyes were closed and from posterior to anterior when the eyes were open in human EEG studies (Alexander et al., 2013; Alamia and VanRullen, 2019), the posterior-to-anterior gradient of peak-alpha frequency remained negative whether the eyes were closed or open (Fig. 2*B*-1*,B*-2). Thus, additional research is needed to assess potential contributions of the posterior-to-anterior gradient to information influx into anterior regions.

We consistently replicated the general negative posterior-to-anterior gradient previously reported with MEG (Mahjoory et al., 2020) using scalp-recorded EEG while participants engaged in a variety of behavioral tasks with their eyes closed, breath focus, vigilance (sustained attention), 2-back (working memory), countdown-by-7 (mental arithmetic), and alternative use (generative thinking) and while they viewed a silent nature video.

A computational model of macaque brain has demonstrated that the posterior-to-anterior hierarchy in temporal integration could arise from a combination of heterogeneity in excitatory connection strengths across regions and specific profiles of long-range connectivity (Chaudhuri et al., 2015). The model further showed that this hierarchy could be dynamically adjusted by differentially gating long-range inputs, raising the possibility that the negative posterior-to-anterior gradient of peak-alpha frequency may be dynamically controlled (also suggested by Mahjoory et al., 2020).

A unique contribution of the current study is the demonstration that the spatial organization of peak-alpha frequency varies between the gradient state where peak-alpha frequency forms a robust negative gradient, diminishing from ∼11 Hz in posterior regions to ∼8 Hz in anterior regions, and the uniform state where it globally converges at ∼11 Hz. Global frequency convergence in alpha-band oscillations may facilitate flexible global communication through phase locking with appropriate phase lags compensating for conduction delays (Chapeton et al., 2019). Thus, alternations between the uniform and gradient states may reflect alternations between promoting flexible global communication and imposing a posterior-to-anterior hierarchy in temporal integration (and potentially also in information flow).

To gain insights into the underlying processes controlling the state variations, we examined the time series of the spatial standard deviation of peak-alpha frequency (*Spatial SD of Freq*) which was monotonically related to the increases and decreases in the negative posterior-to-anterior gradient. The centrally localized first principal component of peak-alpha power (*Pow PC*) driven by posterior regions accounted for nearly 25% of the state variations. Similarly, the anteriorly localized first principal component of peak-alpha frequency (*Freq PC*) accounted for nearly 25% of the state variations. *Pow PC* and *Freq PC* together accounted for nearly 50% of the state variations. These results (though correlational) may suggest that the variations between the uniform and gradient states are controlled by distinct posterior-to-central and anterior processes.

The anterior process, underlying *Freq PC*, may directly control peak-alpha frequency in anterior regions by adjusting temporal integration (e.g., longer integration promoting slower oscillations) through appropriately adjusting the weights of relevant long-range inputs (Chaudhuri et al., 2015). The posterior-to-central process, underlying *Pow PC*, may indirectly control peak-alpha frequency through facilitating synchronization. Specifically, this process may initiate a wave of oscillatory entrainment in the upper alpha band in posterior regions that spreads to central regions to promote global entrainment in the upper alpha band.

Although the anterior *Freq PC* and the posterior–central *Pow PC* approximately additively accounted for the state variations (on average), the correlation between *Freq PC* and *Pow PC* exhibited large individual differences. Notably, *Freq PC*-*Pow PC* correlation modulated how much each variable accounted for the state variations as well as the magnitude of the state variations (in all behavioral conditions except the 2-back and countdown-by-7 conditions). In general, the more positively *Freq PC* and *Pow PC* were correlated, the larger proportions of the state variations they accounted for, and the larger were the state variations. Positive temporal coupling of *Freq PC* and *Pow PC* may facilitate the state variations as follows. Increases in the posterior-to-central synchronization in the upper alpha band (∼11 Hz) may facilitate upward adjustments (from ∼8 to ∼11 Hz) of anterior alpha-band frequencies, thereby promoting a shift toward the uniform state, whereas decreases in the posterior-to-central synchronization in the upper alpha band (∼11 Hz) may facilitate downward adjustments (from ∼11 to ∼8 Hz) of anterior alpha-band frequencies, thereby promoting shifts toward the gradient state.

In the 2-back and countdown-by-7 conditions, *Freq PC*-*Pow PC* correlations were not associated with the magnitude of the state variations or *Freq PC*'s associations with the state variations. We noted that these two conditions required continual engagement of working memory. Working memory has been known to engage alpha-band oscillations in the occipital and parietal regions, likely contributing to memory maintenance by coordinating relevant long-range communication (Roux and Uhlhaas, 2014; Wianda and Ross, 2019) as well as inhibiting distracting information (Bennefond and Jensen, 2012; Roux and Uhlhaas, 2014; Schroeder et al., 2018; Wianda and Ross, 2019). Thus, it is feasible that occipital–parietal alpha-band synchronizations induced by working memory processes played a role in dissociating *Freq PC*-*Pow PC* coupling from the state variations.

In general, it would be interesting to investigate how task-induced alpha-band oscillations interact with the state variations. For example, evidence from visual detection tasks suggests that alpha-band frequency and power in occipital regions play distinct roles in visual perception. Specifically, higher alpha-band frequencies may enable greater visual detection accuracy, likely by increasing sampling rate and processing efficiency, whereas higher alpha-band power may promote the inhibition of irrelevant stimuli as well as perceptual biases driven by expectations (Trajkovic et al., 2023, 2024). It would be interesting to investigate how the state variations, *Freq PC*, and *Pow PC*, and their interrelations, are modulated while performing visual tasks.

Overall, the spatiotemporal dynamics of the state variations (between the uniform and gradient states) were remarkably similar across all six behavioral conditions demonstrated by (1) the similar posterior-to-anterior peak-alpha frequency gradients in the gradient state, (2) the similar broad central localization of *Pow PC*, (3) the similar anterior localization of *Freq PC*, (4) the similar (∼50%) additive accounting of the state variations by *Pow PC* and *Freq PC*, and (5) the similar temporal characteristics of the state variations. It is thus feasible that the state variations reflect general (task independent) mechanisms, which may serve to dynamically balance two modes of information processing: one that facilitates flexible global communication through phase locking in the uniform state and the other that facilitates hierarchical posterior-to-anterior temporal integration (and potentially also anterior influx of information) in the gradient state. Our results, though correlational, suggest that the ongoing state variations are controlled by two distinct processes: an anterior process that directly adjusts peak-alpha frequencies and a posterior process that indirectly adjusts them by increasing or decreasing global entrainment in the upper alpha band. These processes may be coordinated by a slow periodic mechanism with rate-affecting stochastic fluctuations because the state variations in all conditions were characterized by slow oscillations (0.3–0.5 Hz) embedded within random-walk-like fluctuations. Future research is necessary to understand the neural computations underlying the anterior and posterior processes that may control the state variations, their interactions, and their functional roles.

## Synthesis

Reviewing Editor: Anne Keitel, University of Dundee Decisions are customarily a result of the Reviewing Editor and the peer reviewers coming together and discussing their recommendations until a consensus is reached. When revisions are invited, a fact-based synthesis statement explaining their decision and outlining what is needed to prepare a revision will be listed below. The following reviewer(s) agreed to reveal their identity: NONE. Note: If this manuscript was transferred from JNeurosci and a decision was made to accept the manuscript without peer review, a brief statement to this effect will instead be what is listed below.

This revised manuscript was reviewed by one of the original reviewers (for JoN) and the editor. We both agreed that the revisions addressed all concerns. Congratulations on the acceptance of your paper!

Unabridged comments of Reviewer #1:

Advances the Field (Required)

This EEG paper re-examines the gradient of alpha peak frequency from posterior (roughly 11Hz) to anterior (roughly 8Hz) sites, that has recently been reported (Mahjoory et al., eLife, 2020). The results replicate this previous finding and extends it by an interesting observation: the posterior-to-anterior gradient in alpha-peak frequency distribution waxes and wanes in exchange with a more uniform alpha-distribution (at 11Hz) and this occurs independent of task. This adds novel information to emerging evidence of dissociated processes regarding alpha-frequency and -amplitude.

Comments to the Authors (Required)

I have reviewed this paper before for another journal. My comments have been transferred to eNeuro and have all been adequately addressed. This is a well written and well documented paper. I appreciate the specifications and more careful interpretations provided in the revisions. I have no further points.
