# Supplementary figures and images for: Dynamic Formation of a Posterior-to-Anterior Peak-Alpha-Frequency Gradient Driven by Two Distinct Processes
Source: eNeuro. 2024 Aug 23;11(8):ENEURO.0273-24.2024. doi: 10.1523/ENEURO.0273-24.2024 (PMC11373881; doi:10.1523/ENEURO.0273-24.2024)

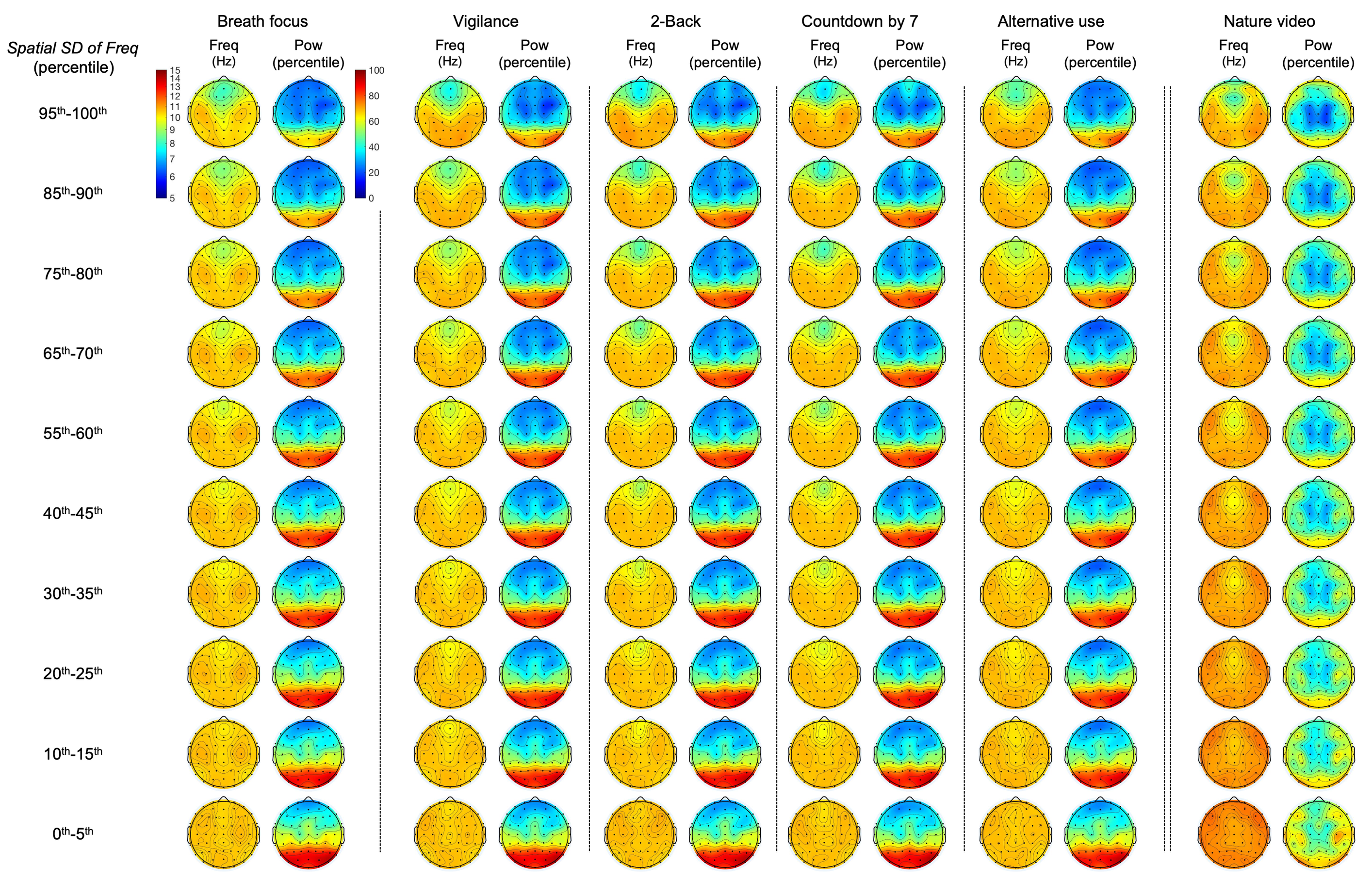

Supplement: Figure 2-1 — Spatial patterns of peak-alpha frequency (left columns) and peak-alpha power (right columns) at different percentile levels of the spatial standard deviation of peak-alpha frequency (Spatial SD of Freq) for the six behavioral conditions (column pairs). The spatial patterns are shown for ten evenly spaced 5% intervals of Spatial SD of Freq. The lowest and the highest levels correspond to the “uniform” and “gradient” states, respectively, shown in Figure 2B-2 and 2B-3. Note that as Spatial SD of Freq increased, the negative posterior-to-anterior gradient of peak-alpha frequency gradually emerged and strengthened without going through any distinct intermediate patterns. This indicates that Spatial SD of Freq (spatial standard deviation of peak-alpha frequency) provides an appropriate measure to quantify and track the variations between the uniform and gradient states. Download Figure 2-1, TIF file. [file eneuro-11-ENEURO.0273-24.2024-s001.tif]
